# Self-Weighted Multilateration for Indoor Positioning Systems

**DOI:** 10.3390/s19040872

**Published:** 2019-02-20

**Authors:** Alberto Fornaser, Luca Maule, Alessandro Luchetti, Paolo Bosetti, Mariolino De Cecco

**Affiliations:** Department of Industrial Engineering, University of Trento, Via Sommarive 9, 38123 Trento, Italy; luca.maule@unitn.it (L.M.); alessandro.luchetti@unitn.it (A.L.); paolo.bosetti@unitn.it (P.B.); mariolino.dececco@unitn.it (M.D.C.)

**Keywords:** multilateration, ultra-wide-band, indoor localization, measurement, uncertainty

## Abstract

The paper proposes an improved method for calculating the position of a movable tag whose distance to a (redundant) set of fixed beacons is measured by some suitable physical principle (typically ultra wide band or ultrasound propagation). The method is based on the multilateration technique, where the contribution of each individual beacon is weighed on the basis of a recurring, self-supported calibration of the measurement repeatability of each beacon at a given distance range. The work outlines the method and its implementation, and shows the improvement in measurement quality with respect to the results of a commercial Ultra-Wide-Band (UWB) system when tested on the same set of raw beacon-to-tag distances. Two versions of the algorithm are proposed: one-dimensional, or isotropic, and 3D. With respect to the standard approach, the isotropic solution managed to reduce the maximum localization error by around 25%, with a maximum error of 0.60 m, while the 3D version manages to improve even further the localization accuracy, with a maximum error of 0.45 m.

## 1. Introduction

The indoor localization of objects, statics or dynamics, is a topic of high interest for the strong technological implications deriving from it. Having a GPS like technology for indoor applications would indeed lead to new and various automation challenges: robotics, manufacturing and logistics for the industrial field, but also for health care, domestic management and much more.

In the last decade, suitable technologies, both in terms of technological readiness and affordability for the potential users, were born and began to be available on the market. Most of those are based on distance measurements, multilateration, between beacons relying either on UWB antennas or on ultrasound emitters/detectors [1,2]. Such systems are today available as relatively low-cost solutions, also in the form of kits.

For radio-based systems, such as UWB (see for example [2]), each element provides both measurement and networked communication, as well as on-board computational resources even if limited. For ultrasound-based systems (like for “Marvelmind robotics,” http://www.marvelmind.com), the ultrasound only provides distance measurement and there is typically a digital radio channel used for time synchronization and for data exchange. As for UWB systems, computational resources of a microcontroller are available per beacons as well.

The typical configuration foresees four, or more for redundancy, fixed beacons plus movable tags, the ones that have to be fastened to or embedded with the objects to track/identify in space. The 3D position of the beacons is usually obtained through a calibration process—for the position of tags, the data of interest, which is assessed on the basis of each Beacon to each Tag Distance (BTD) measurements by means of a multilateration algorithm.

Main advantages consist of the identification of each tag, with the possibility to easily embed tags within objects/subjects [3] and in the tracking or in their combination with other sensors, usually inertial, for a more reliable and accurate assessment of motion, especially for indoor environments [4,5]. In many cases [6], the measurement of the tag position is reinforced by applying a weighted multilateration position estimation based on a Received Signal Strength Indicator (RSSI) with respect to each tag. Although this approach proved effective, sometimes an RSSI might not be available, or there is no clear evidence of a strong correlation between RSSI and *precision* of BTD measurements [6], such as using ultrasound beacons.

This work presents then a new approach to the multilateration practice. A weighting factor is calculated on the basis of the previous history of BTD measurements for a given beacon at a given distance range rather than applying a compensation on only the distance calculated by multilateration and RSSI. An elastic method used in combination with the weighting factor resulting from the uncertainty level and the recursive update based on the history of measurements and locations. An experimental dataset collected in a controlled and calibrated drone arena was used as reference related to the assessment of the metrological performances of the proposed solution with respect to the original algorithm associated with the considered hardware.

## 2. State-of-the-Art

Recently, UWB applications are widely employed starting from the communications on the radio frequency channel but also for the indoor localization. Historically, UWB technology was restricted to military applications under classified programs such as highly secure communications. The Federal Communication Commission (FCC) standardized in 2002 the usage of the UWB system for data communication and safety under the strict rule on the frequency and the maximum power allowed [7]. Nowadays, UWB represents a very promising technology also in the field of indoor localization due to the high precision and accuracy in the measurement of the pulses generated: modern UWB localization systems reach an accuracy of about few centimeters.

The accuracy of radio-based localization systems, especially in indoor applications, is affected by the multipath interface, usually due to reflections or echoes of the signal caused by walls, floor, and ceiling. UWB systems are less affected by the aforementioned situation [8] and, due to the low power and the wide spectrum (from a very low value of frequency), they can coexist with other radio technologies and can penetrate different materials including walls, bodies, or other obstacles in general.

Parameters like Transmitter-Receiver (Tx-Rx) phase noise or high Rx sampling rate affects the overall system accuracy. Ref. [9] simulates a complex UWB localization system considering the largest number of these parameters, showing the possibility of obtaining an accuracy in the millimeter-range inside a dense environment. In [8], the authors propose a low-cost positioning system for the indoor application that reaches in this case a sub-centimeter accuracy using a time domain measurement. Despite a lower accuracy, such value results in being more than sufficient for most of the applications in which the localization of a tag is required. Ref. [10] proposes a method to increase the position accuracy by considering the time measurement uncertainty and describing the unwanted influence of the antennas and other external components that have an impact on the measure.

This work presents an innovative technique to improve the accuracy of a low-cost UWB localization system. The position estimation is determined by calculating a weighed factor based on the previous measurement. The correction is based on the uncertainty estimation of the measure by using the RSSI information to compensate the calculated distance of each BTD. Compared to the state of the art, the environmental factors like surrounding objects as well as the topology of the localization system are neglected, focusing instead on the metrological performances of the localization algorithm. Of course, the surrounding environment has a strong impact on the performance of a UWB localization system, and densely cluttered environments decrease the quality of the measurement due to the partial or complete line-of-sight blockage [11].

## 3. Method

The multilateration system is made by *N beacons*, assumed fixed in space, located at known, calibrated, coordinates $\left\{x_{k},y_{k},z_{k}\right\}$. A movable *tag* is instead located at an unknown position {X,Y,Z}. The system is over-determined, and the position of the tag can be calculated by zeroing the pseudo-energy associated with the BTDs.

For the *k*-th beacon, the BTD strain energy $E_{k}$ with respect to the tag can be expressed as [12,13]:

(1) $$E_{k}=\frac{{\left(X-x_{k}\right)}^{2}+{\left(Y-y_{k}\right)}^{2}+{\left(Z-z_{k}\right)}^{2}}{r_{k}^{2}}-1-2\;e_{k},$$

where $r_{k}$ is the BTD measured by the *k*-th beacon, and $e_{k}$ is its strain. The multilateration can be calculated by solving the constrained minimum problem that minimizes the individual strain $e_{k}$, under the condition represented by $E_{k}=0$. Because of its low computational cost and ease of implementation, the Newton’s method is mostly used for such a numeric solution.

Given the general formulation, this is modified accordingly to the fact that each beacon behaves differently depending on the distance of the tag and the environmental conditions. The strain $e_{k}$ is then usually weighted by a suitable function $W_{k}\left(r_{k}\right)$ of the raw distance $r_{k}$, thus leading to the Lagrange function:

(2) $$L=\sum_{k=1}^{N}-e_{k}/W_{k}\left(r_{k}\right)-\lambda_{k}E_{k}.$$

In principle, the weight functions $W_{k}$ have to be close to 1 when the BTD measurement is highly reliable, and get larger as the reliability decreases.

### 3.1. Elastic Weighted Multilateration

The novelty of the proposed approach stands in the way in which the weighting function is calculated.

**Figure 1:**
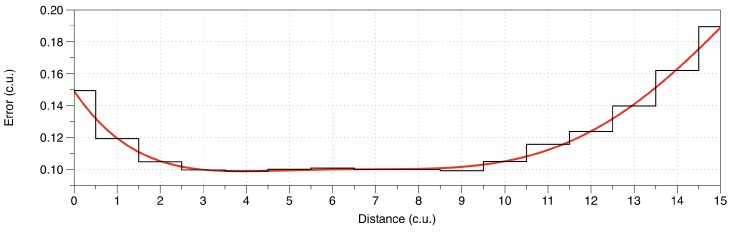
Example of random error data for a single beacon-tag pair. The red line is the hypothetical behavior; black stepped line is the result of a binned running standard deviation.

**Figure 2:**
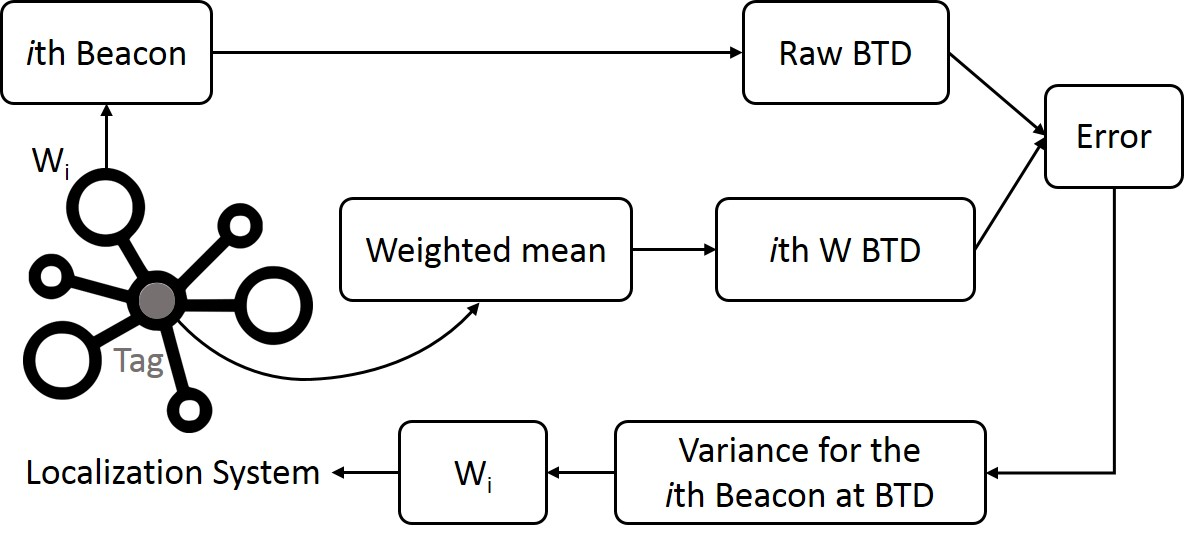
Schematic representation of the proposed trilateration algorithm. The logic represents the processing loop for the *i*th beacon at a new frame acquired by the system.

Rather than being based on an instant random value as the RSSI (which, being a random variable, tends to increase measurement noise), the weights are evaluated based on the running variance of previous BTD *deviations*, as the differences between the measured BTDs and those calculated by the self-weighted multilateration method itself, for each beacon within a small range around the current position, Figure 1. In other words, at every step, the current raw BTD value, measured by each single beacon, is compared to the distance calculated by the simple weighted mean, and the resulting BTD *deviation* is used for updating the BTD deviation variance in the current bin. Figure 2 reports a schematic representation of the process.

More specifically, Equation (2) needs weights in the form $W_{k}\left(r_{k}\right)$, meaning that, for the *k*-th beacon, the weight values have to be defined as a function of the current BTD measurement ($r_{k}$). This can be provided by collecting BTD deviations within the same bins as the system collects measurements, and by applying the formulas for calculating the statistics to each updated bin and for each beacon. It is worth noting that the BTD deviation statistics, mean and variance, shall be calculated by using recurring formulas, in order to minimize the memory footprint.

In the current implementation, the distance bins are defined by dividing the maximum range for a sufficiently large number of bins, here set to 40: the value was selected as a trade-off among the typical memory amount available onboard and the desired spatial resolution for localization. The maximum range itself is defined as 1.5 times the maximum inter-beacon distance, from the operative gold standard that suggests designing the system to have the movable tag within such a distance value.

The actual weights are then calculated on the basis of the binned sample variance $s^{2}$ as follows:

(3) $$W_{k}\left(r_{k}\right)=\left\{\begin{array}{cc} 1, & s_{k}^{2}\leq r_{0}^{2},\\ (s_{k}^{2}-r_{0}^{2})b+1, & \mathrm{otherwise}, \end{array}\right.$$

where *b* is a gain factor to be calibrated, whose actual value makes the weighting more effective when it is larger than 1.

Given that the beacon and tag firmware typically runs on low-power systems that have limited memory and computational power, it is necessary to care about the definition of the algorithm used for calculating the recursive statistics, and in particular the sample variance $s^{2}$. A recursion formula modified with respect to the more common one is reported; the latter contains differences between similar numbers and thus suffers from rounding errors when run on single precision CPUs:

(4) $$s_{1}^{2}:=0;\;s_{n}^{2}=\frac{1}{n-1}z\left(\left(n-2\right)s_{n-1}^{2}+\frac{n}{n-1}{\left({\bar{x}}_{n}-x_{n}\right)}^{2}\right),$$

where $x_{n}$ is the *n*-th BTD measurement, and ${\bar{x}}_{n}$ is the running average after *n* BTD measurements, which in turn may be calculated by a recursion formula:

(5) $${\bar{x}}_{1}:=x_{1};\;{\bar{x}}_{n}=\frac{1}{n}\left(\left(n-1\right){\bar{x}}_{n-1}+x_{n}\right).$$

The application of these schemes requires a minimum amount of memory, so the current values ${\bar{x}}_{n}$ and $s_{n}$ can be stored on each beacon for resiliency of operations.

### 3.2. Three-Dimensional Space

The previous formulation provides a one-dimensional, spherical isotropic, assessment of measurement uncertainty, for each beacon. Such approximation can be extended to the 3D domain by applying minor modifications to the algorithm.

Instead of a linear binning vector, a voxelized volume is defined for the entire system and independently assigned to each beacon. The volume’s extremities are defined accordingly to the maximum operative ranges, from the maximums beacon’ range and their relative positions. The voxel structure is organized as a 3D matrix in which each cell, identified by the triplet of indices ijk, contains the 3D position that defines the center of the voxel bin.

The extension to the 3D is achieved by substituting the selection of $s^{2}$ from the linear vector, and thus a mapping of a distance into a single index, with the selection of a cell from a 3D matrix. The operation requires two steps, reported in Algorithm 1:

1. the initial resolution of the trilateration using the one-dimensional notation. The incremental information collected in the process remain valid for a small motion of the tag, for such a reason the online calculation of the parameters provides already a reliable assessment on the position of the tag in the 3D space.
2. given the first guess solution for the 3D position, the algorithm initially extracts the stored $s^{2}$ from the closest voxel bin of the 3D voxel matrix, from the center contained in the ijk-th cell, and then a refinement of the position is achieved by a second optimization. The resulting values of $s^{2}$ and $W_{k}$ are updated and are stored back in the cell.

**Algorithm 1:** 3D trilateration
**1** new data from N beacons;**2** trilateration with the isotropic formulation and the on-line parameters from the previous step;**3** identification of closest voxel bin of the 3D voxel volume(matrix), at the *ijk* indices, from the solved *xyz* position;**4** update of $s^{2}$ and Ws;**5** trilateration with updated parameters;**6** update the *ijk* cell of the voxel volume;


The trade-off among the one-dimensional and the 3D notation is in the memory required to run the algorithm. In the first, very little memory is required. The algorithm can be run on the same electronics that constitute the beacon, usually constituted by a low-power micro controller, few megabytes of internal memory and an antenna. No mapping is possible given the isotropic spatial behaviour resulting from such formulation.

As for the latter, a higher amount of memory is required, depending on the operative volume’s dimensions and the voxels’ spatial resolution. A much more complex hardware should therefore be paired to each beacon. The advantage is, however, the direct mapping of areas affected by a higher localization uncertainty, for each beacon, directly in the 3D operative space.

Given the higher detail in the modelling of uncertainties in space, as a further advantage, the 3D extension should achieve a more accurate localization than the isotropic version of the algorithm.

## 4. Experimental Testing

The proposed computation framework was implemented in MATLAB for testing purposes. This can elaborate the position of the movable tag given its distances with respect to an arbitrary number of beacons.

**Figure 3:**
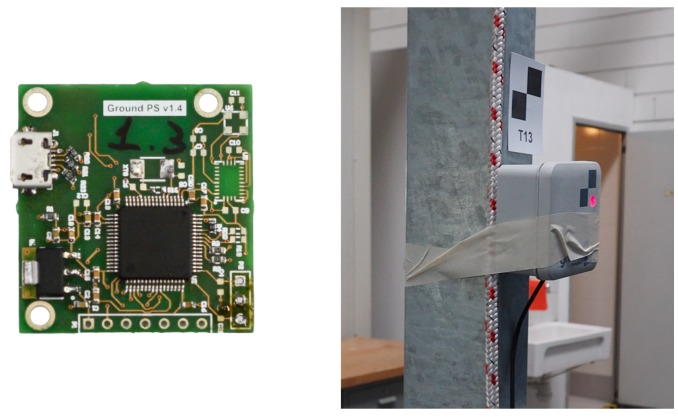
The considered beacon hardware and the experimental setup. The marker on the box represents the reference point for the calibration of the system.

The prototype software has been run on a dataset of BTD measurements collected by a UWB system made of six fixed beacons and one movable tag. The system is manufactured by GroundPS (http://groundps.com), Figure 3 and it is primarily designed as a drop-in replacement of GPS navigation for UAVs. It has a maximum rated BTD of 200 m, a mass of 20 g, and physical dimensions of 30 mm × 35 mm × 10 mm. The technical specifications state a 3D positioning precision lower than 100 mm.

**Figure 4:**
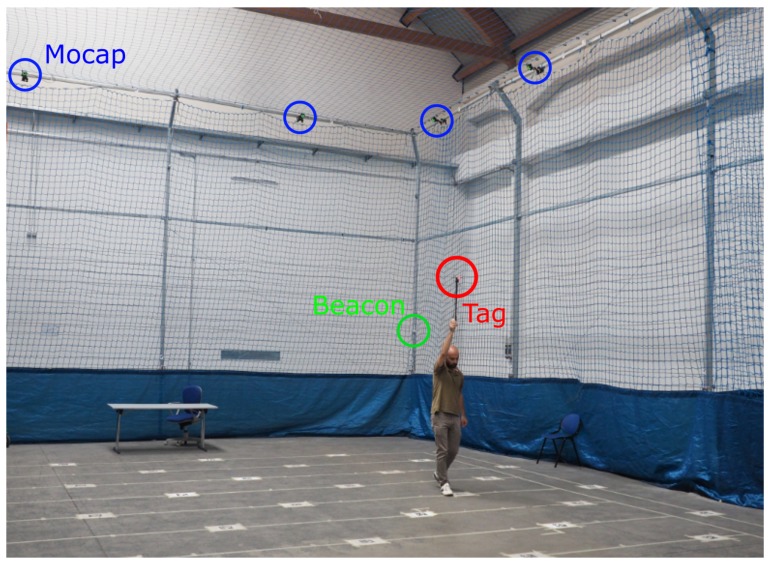
The experimental setup exploited for data collection. In the picture are visible: in red, the moveable tag on the top of the stick while being moved by hand inside the operative space, in blue, the motion capture system (Mocap) used to measure the reference position of the tag, and in greed, one of the eight beacons.

The original dataset was collected at a sample rate of 50 Hz by manually moving the tag along a combination of random and straight motions, the latter by following ground lines, Figure 4. The tag was moved on top of a 2 m pole, so that the expected values for the *z* coordinates fall within the 0–4.5 m range.

The dataset also contains the 3D coordinates as calculated by a standard algorithm, proprietary to the system manufacturer.

A 12-camera motion capture system(Mocap), OptiTrack Prime, was used as reference instrumentation for the measurement of the ground-truth (the nominal accuracy is sub-millimetric). Three infrared passive tags were attached to the UWB tag for its tracking in the operative space. The motion capture system and beacons’ positions were calibrated to achieve the homogeneous representation of measured positions.

## 5. Results

### 5.1. Time-Domain Analysis

**Figure 5:**
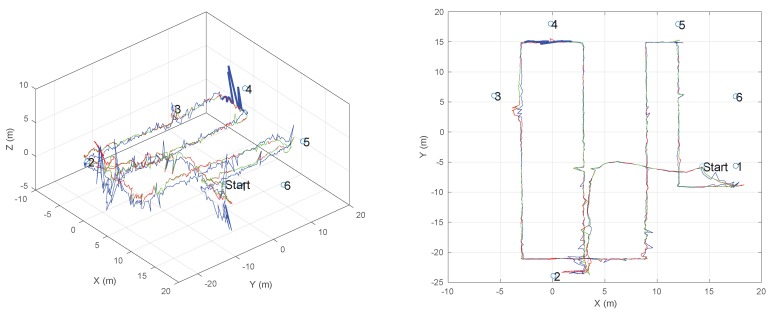
Trace of test measurement (left in 3D, right is top view): blue line is the original trace as computed by the original manufacturer’s software; red trace is the proposed solution, with initial, non-calibrated statistics (*first-run*); green trace is the proposed solution, using the calibrated statistic at the end of the previous lap (*second-run*). Anchors’ positions are represented by circles, marked with numbers 1–8.

**Figure 6:**
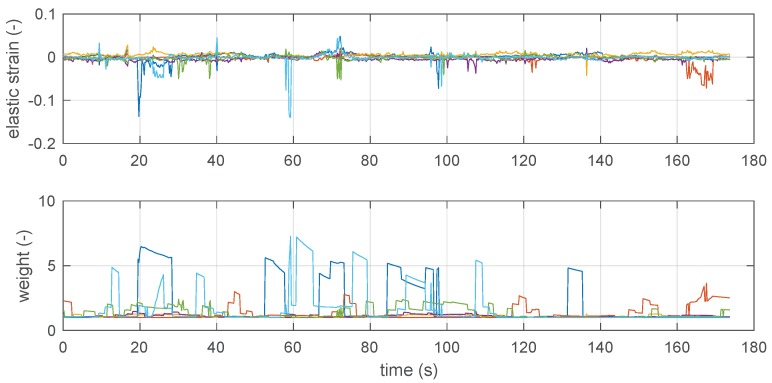
*Top*: Elastic strain associated to beacon 1–6, as calculated by the optimization algorithm, as a function of time along the test measurement of Figure 5 (during second-run). *Bottom*: Evolution of weights applied during the second-run.

Figure 5 reports the trace measured by the system with the manufacturer’s reference algorithm (blue line) and the traces calculated with the proposed algorithm on the basis of the same set of raw BTD measurements. The red line represents the trace calculated by using freshly initialized running statistics (i.e., index 1 in Equations (4) and (5)), while the green line represents the result of running the algorithm against the same dataset, but keeping the running statistics at the end of the first lap. The same Figure 5 also reports the positions of the six fixed tags, represented by numbered blue circles. Figure 6 reports both the elastic strain and the weights assigned by the algorithm during the second passage in the area. The spikes in the weight highlight those beacons that were affected by a high error in trilateration, and thus a high uncertainty. These played therefore a less relevant role in the localization strategy.

**Figure 7:**
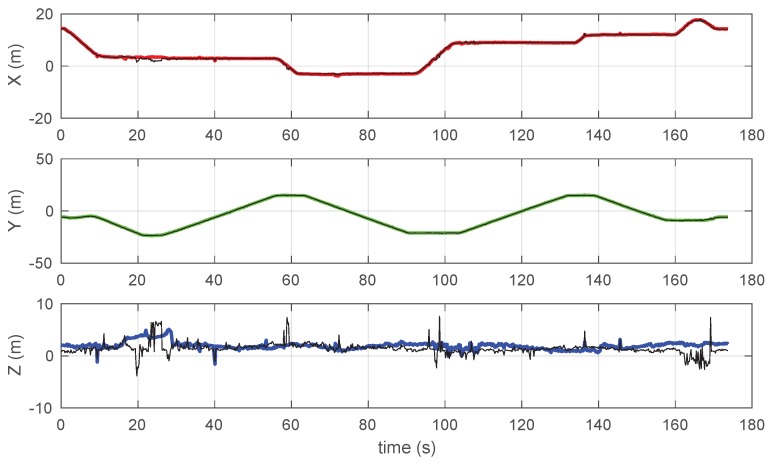
Coordinate vs. time profiles for the test measurement reported in Figure 5: manufacturer’s algorithm data are reported in black.

It is evident that the proposed algorithm gives a smoother and more regular trace, effectively rejecting the larger spikes in the *z*-direction of the original profile. It is worth noting that the predominance of random spikes in the *z*-direction results from the beacons being much less sparse in the *z*-direction than along the other two directions. The individual coordinate vs. on the time charts, reported in Figure 7, allows for further appreciating the difference. In particular, the *x*-chart shows that the black line (manufacturer’s algorithm) appears to be more noisy on a small scale, while the *z*-chart also shows as the proposed system rejects some spikes (see for example around 25 s and 59 s) and, in particular, it has a very limited number of events at negative *z*, which correspond to unfeasible underground positions.

When Figure 6 and Figure 7 are observed together, the coordinate vs. on the time charts of Figure 7 allows for justifying better resilience of the proposed method to random jerks due to measurement noise of individual BTD measurements. For example, it may be observed that the prominent jerks in *z*, observed at 59 s, 135 s, and 165 s only affect the strain of one single tag; furthermore, the corresponding weights are significantly larger than 1 in the same conditions so the weighted algorithm effectively rules out the contribution of the noisy tag, returning a much more stable output.

**Figure 8:**
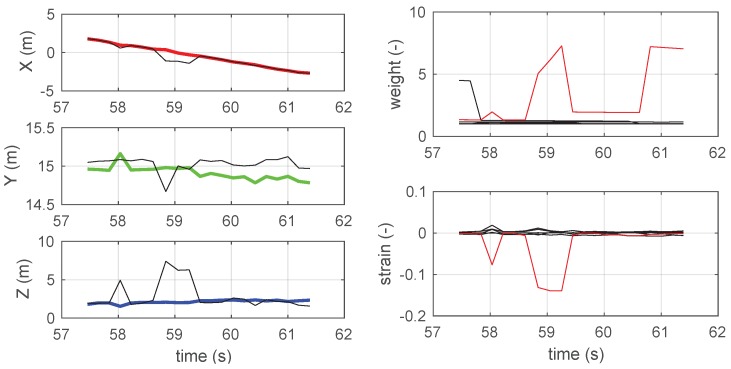
*Left*: Coordinates detail around the spike in the original trace (blue), which is marked with a thicker line in Figure 5 (close to anchor 4). *Right*: Strain and weight detail around the spike in the original trace (blue), which is marked with a thicker line in Figure 5 (close to anchor 4). The red line is related to anchor 6; other anchors are black.

The details reported in Figure 8 allow for better appreciating this effect: the first figure shows that the jerks, appearing between 58.5 s and 59.5 s in the manufacturer’s reconstruction, are successfully removed by the proposed algorithm, thanks to the fact that in the same time interval the single large value of the sixth beacon suppresses the otherwise large strain value.

In other conditions, for example around 72 s and 100 s, there is more than one single noisy beacon, or more than one single beacon having low reliability in that configuration, so that the resulting 3D position is still affected by significant noise. Nevertheless, it is evident that a more redundant beacon configuration (i.e., more than six beacons) is likely more resilient to these types of issues.

**Figure 9:**
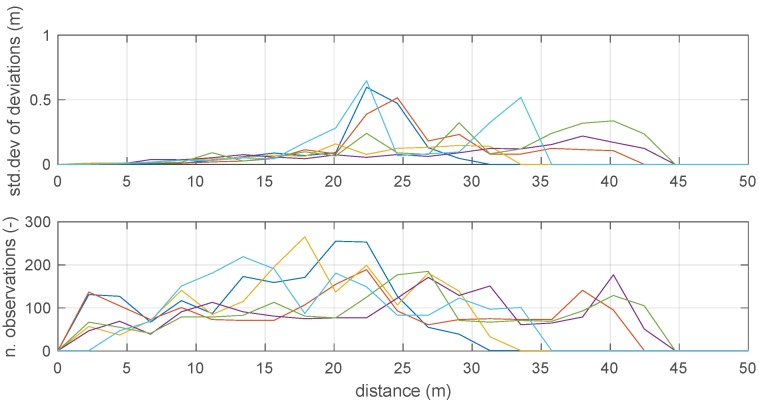
Standard deviation of measurement distance deviations at different distance ranges for the six anchors. The binning is defined by dividing the measurement range into 40 bins.

The two charts in Figure 9 present the binned standard deviation of BTD measurement deviations obtained by the running statistics for the six beacons, as a function of the measured distance and at the end of the first run. The same figure also shows the number of collected observations per bin, which is typically large enough to assume a reliable estimate of the statistics. As expected, the standard deviation tends to increase with distance. Some of the beacons, though, show a remarkably different trend. This is consistent with the observation by the system manufacturer and from literature [14] it is found that the distance measurement is often dependent on the spatial position, and not only on the beacon-tag distance. Consequently, further improvements in the weighting technique could be obtained by switching from a one-dimensional binning (standard deviation vs. distance) to the 3D binning (voxels).

Finally, the presented self-weighted algorithm has been run against a static tag, placed on a stable position in the central zone of the measurement area and collecting 155 points. The 3D position, calculated on the 155 BTD measurements, presents the following standard deviations:

- **manufacturer’s algorithm:**$s_{x}=0.034\;m,s_{y}=0.022\;m,s_{z}=0.480\;m,$
- **self-weighted algorithm:**$s_{x}=0.028\;m,s_{y}=0.020\;m,s_{z}=0.047\;m,$

which confirms that the proposed algorithm ensures a significantly improved repeatability of the measured 3D coordinates, especially along the vertical direction where the range of vertical distances between the tags is minimal, in the test configuration and also in a typical configuration.

### 5.2. Frequency-Domain Analysis

The trajectories of Figure 5 have been transformed in the frequency domain in order to show that the speed signal along the trajectory, resulting from the self-weighted multilateration approach, is more consistent than the speed signal resulting from the manufacturer’s multilateration. This is due to the relatively stable and low-acceleration motion resulting from manually moving the beacon during the test.

**Figure 10:**
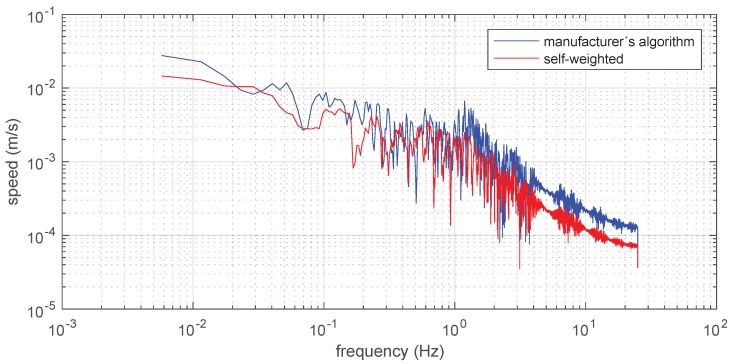
Discrete Fast Fourier Transform of the speed vs. time signal obtained by the test trajectory.

In both cases, the speed signal has been determined after calculating the distances between consecutive points and also after re-sampling the resulting data to 50 Hz, in order to take care of less than ten missing points. The frequency spectrum of speed along the whole trajectory has been obtained by discrete Fast-Fourier-Transform and it is reported in Figure 10 for both self-weighted and manufacturer’s multilateration.

It is evident that the multilateration presented here shows a significantly reduced speed and jerkiness of motion, which is more compatible with the way the data have been collected, for example by manually moving the tag attached to a 2 m pole, especially above 7 Hz, which is the typical human actuation limit [15].

**Figure 11:**
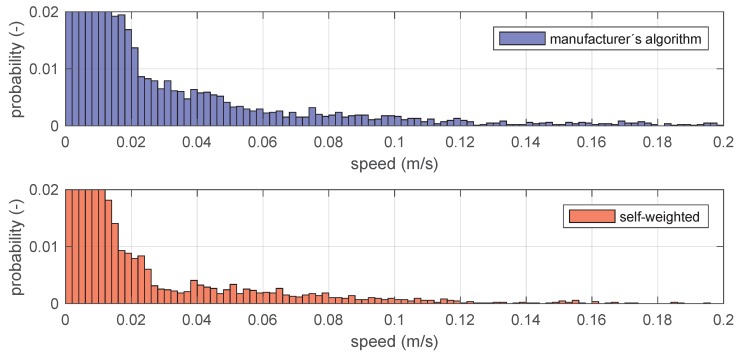
Speed distribution along the test trajectory.

**Figure 12:**
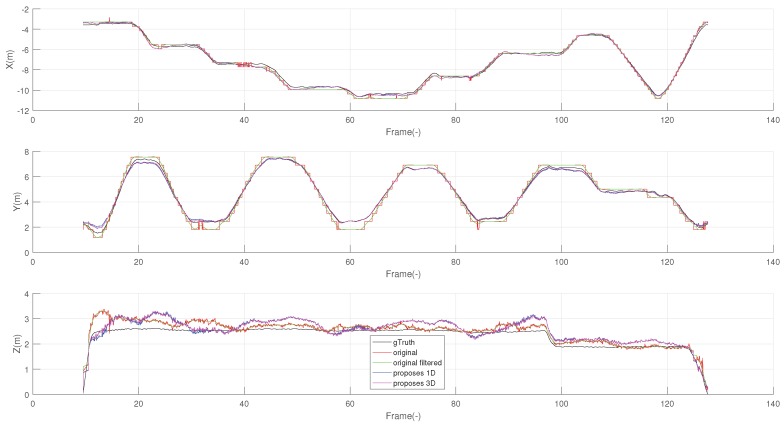
Comparison of all methods divided among the xyz coordinates. The one-dimensional version in blue, 3D in magenta, the default version in red, this last filtered in green, and the ground truth reference in black.

Finally, Figure 11 also reports the distribution of speeds in the range 0.02–0.2 m/s. Consistently with the above concept, a significantly larger probability for higher speed confirms that the speed signal, resulting from the manufacturer’s algorithm, is noisier than that resulting for the proposed self-weighted multilateration algorithm. The same can be appreciated from Figure 12, which reports the xyz components independently. The *z* coordinate is the one affected by the highest signal-noise-ration, from the positioning of the beacons. As for *x* and *y*, these achieved from both proposed solutions a smoother trend in localization, closer to the real motion imposed. This generates minor spikes in the velocity, and thus less components at a high frequency in the frequency domain.

### 5.3. Computation Performances

The MATLAB implementation can elaborate the position of the moveable tag given its distances respect to an arbitrary number of beacons. It can process about 90 localizations per second on a 2.6 GHz desktop class CPU. A first porting of the algorithms to C improved this feature of around 10 times, so it is reasonable to attain better than 50 Hz rate on a 32-bit embedded, low-power processor.

### 5.4. Three-Dimensional Maps

**Figure 13:**
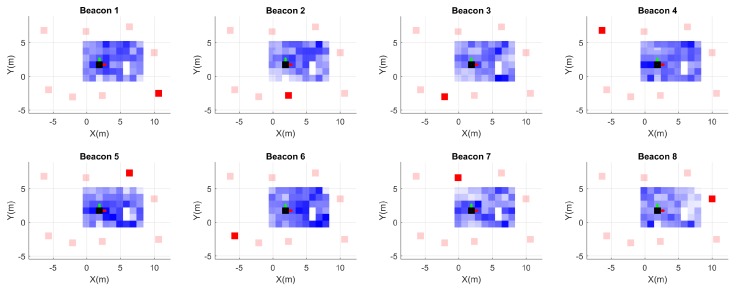
Voxel 3D maps for each beacon (anchor). The gradient in the color represents the $s^{2}$ level in the voxel, 1 m spatial resolution; the reference beacon for the map is in dark red.

Figure 13 reports the results achieved with the 3D version of the algorithm, the voxelized map for each beacon. The test volume was set with a voxelization of 1 m spatial resolution. Voxels further from the beacon achieve a higher $s^{2}$ value and thus play a less relevant role in the trilateration.

**Figure 14:**
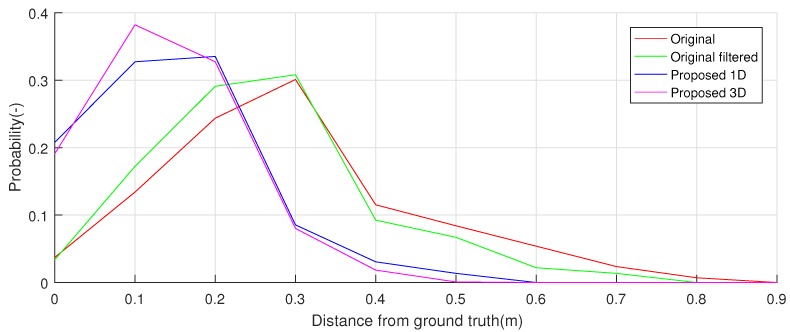
Error of the ground-truth reference with respect to the possible localization strategies. The 3D version of the proposed method is the solution that achieved the minor error and thus the best localization performances.

Figure 14 reports the error histograms between the ground truth and the localization achieved with the standard software and the proposed solution, both one-dimensional and 3D. The proposed solution achieved already with the one-dimensional version a better localization than the standard approach, with an improvement in the reduction of the maximum errors of round 25%, moving from an error of 0.8 m to 0.6 m. As for the 3D version, this achieved a further improvement with respect to the one-dimensional version, quantifiable in 25% and a maximum error of 0.45 m.

In addition, the hypothesis testing on collected data confirmed the marked difference among the considered localization strategies: the paired *t*-test (the log10 of the localization error was taken as a factor, that to achieve the normality assumption of data) among the isotropic version of the algorithm and the standard one achieved a *p*-value close to 0. As for the comparison of the isotropic and the 3D version, this achieved a *p*-value of 5.06%, an index of a marginal difference in localization.

## 6. Discussion

The experimental results highlighted that the proposed solution achieves a better localization accuracy than the standard approach, with measures affected by a lower bias and spatial jumps. Such elements result in being particularly evident if it is considered only the *xy* planar motion of the tag. Still some discrepancies remain in the vertical direction, for both approaches, probably due to relative disposition of beacons in the considered hardware setup.

As for the computational performance of the two versions of the algorithm, the trade-off relies on the hardware requirements in terms of memory and computational power: the 3D version of the algorithm requires more than three times the amount of memory and twice as much processing as the one-dimensional one; however, with this solution, it is possible to generate a discrete map in which each bin contains information about the localization uncertainty for that specific 3D spot. Depending on the operative scenario, application field and hardware configuration, each of these versions may result in being more preferable than the other.

The limitations of the proposed analysis come from the considered operative space, which can be considered ideal for this kind of application, free from echoes and obstacles. These elements usually lower the localization accuracy of radio systems and are typical of an indoor operative scenario, like a building. Such a condition was not considered in the proposed work because of the nature of the testing environment, more similar to a warehouse, and the objective of the analysis: the initial verification of the capabilities of a self-weighting trilateration based on the same localization uncertainty. More investigations are needed in order to further analyze the behaviour of the algorithm in different, and more complex, scenarios. Future lines of investigation will focus then on (i) the collection of a wider dataset, considering drones for the automatic path generation and execution, (ii) the introduction of some physical obstacles inside the operative area will be considered and thus verify the capability of the 3D mapping in identifying these from the variance of the localization. Such a functionality would be useful for the automatic identification of indoor safe areas for autonomous drone operations after an initial mapping of the environment.

## 7. Conclusions

The proposed solution aims at reinforcing the multilateration position estimation by weighting the individual BTD measurements with the known precision of each sensor in its given distance range. The latter position is progressively updated thanks to recursive estimation of the variance of the measurement deviation, which is defined as the difference between the BTD measurements and the beacon-tag distance as calculated by the multilateration itself. In essence, the presented approach, while assuming a calibrated system for compensating systematic errors, exploits redundancy of measurements for reducing random errors.

Two versions of the algorithm were proposed, one-dimensional, or isotropic, and three-dimensional. The maximum localization error, with respect to the standard approach, resulted in a reduction of around 25% for the one-dimensional case (maximum error of 0.60 m), while the 3D version managed to improve even further the localization accuracy, reaching a maximum error of 0.45 m, which represents a suitable value for the typical application of the UWB technology.
